# Epithelial salivary gland neoplasms in pediatric patients: A comprehensive review

**DOI:** 10.4317/medoral.26983

**Published:** 2025-04-06

**Authors:** Maria Eduarda Pérez-de-Oliveira, Sebastião Silvério de Sousa-Neto, Pablo Agustin Vargas

**Affiliations:** 1 DDS, MSc, PhD, Department of Clinical and Preventive Dentistry, Federal University of Pernambuco, Recife, Pernambuco, Brazil; 2 DDS, MSc, Department of Oral Diagnosis, Piracicaba Dental School, State University of Campinas, Piracicaba, São Paulo, Brazil; 3 DDS, PhD, FRCPath, Department of Oral Diagnosis, Piracicaba Dental School, State University of Campinas, Piracicaba, São Paulo, Brazil

## Abstract

**Background:**

Salivary gland tumors (SGTs) are a rare group of neoplasms in pediatric patients, characterized by diverse histological subtypes and distinct biological behaviors. This manuscript aims to provide a comprehensive overview of the demographic, clinical, and histopathological characteristics of SGTs in this population, with the goal of enhancing understanding of their presentation and implications for treatment.

**Material and Methods:**

An updated descriptive literature review was performed to identify studies reporting the clinicopathological features of epithelial SGTs in pediatric patients.

**Results:**

SGTs in pediatric patients are slightly more prevalent in females and typically arise during the second decade of life. The parotid gland is the most commonly affected site for both benign and malignant tumors, which usually present as an asymptomatic mass. Pleomorphic adenoma and mucoepidermoid carcinoma are the most frequently observed entities. Surgical intervention remains the primary treatment modality, and the overall prognosis is generally favorable.

**Conclusions:**

This review highlights that SGTs in pediatric patients present distinct clinical features and prognostic outcomes compared to adults. Although they are rare in this age group, clinicians should remain vigilant to these neoplasms when assessing nodular masses in both major and minor salivary glands.

** Key words:**Salivary gland tumors, pediatric, mucoepidermoid carcinoma, pleomorphic adenoma.

## Introduction

Salivary gland tumors (SGTs) are a group of lesions with a wide range of histological subtypes and distinct biological behaviors representing a diagnostic and therapeutic challenges for both pathologists and surgeons (1). The incidence of SGTs varies by country, but they are generally rare, with an annual incidence of approximately 2.5 to 3.0 per 100,000 people (2). Malignant SGTs comprise 2-6% of all head and neck cancers with an annual worldwide incidence of 0.07% corresponding to 54,799 cases per year (3).

Overall, SGTs have a slight female predilection with a peak of incidence between the fourth to seventh decades of life. Parotid gland is generally the most affected site, followed by minor salivary glands, and submandibular glands, comprising about 60%, 30%, and 8%, respectively. Sublingual tumors are extremely uncommon. More than half of the minor gland tumors are likely to be malignant. Tumors involving minor glands have worse prognosis, higher recurrence rate and poor outcomes compared to major gland tumors. Other sites for SGTs include the larynx, trachea, lacrimal glands, nasal cavity, and heterotopic salivary tissue within the mandible and the lymph nodes (1,4,5).

Clinically, SGTs in adults typically manifest as a painless swelling. Both benign and malignant lesions may exhibit similar clinical features; however, certain characteristics - such as the progression, presence of pain, and ulceration - can help distinguish benign from malignant conditions (1,4-6). According to Mariz *et al* (6), changes in color and the presence of pain are associated with a malignant diagnosis in the palate.

The most prevalent benign SGT is pleomorphic adenoma (PA), while the most frequently encountered malignant SGT is mucoepidermoid carcinoma (MEC) (1,4,5). However, some studies have identified adenoid cystic carcinoma (ACC) as the most common malignant variant in some geographical areas, including Africa, Finland, and Israel (5,7,8). Alsanie *et al* (5) conducted a multi-center evaluation worldwide, analyzing a total of 5,739 cases. Their findings revealed that 65% of the tumors were benign, while 35% were malignant. The most commonly encountered benign SGT was PA, followed by Warthin tumor and basal cell adenoma. In contrast, the most frequently observed malignant tumors were MEC, ACC, and polymorphous adenocarcinoma (5).

SGTs in pediatric patients can present distinct clinical features and prognostic outcomes compared to those in adults. Therefore, this review aims to provide a comprehensive overview of the key aspects of primary epithelial SGTs in this age group. This study is part of a special issue featuring presentations from the X International Symposium on Advances in Oral Cancer, held in Bilbao, Spain, in July 2024.

## Material and Methods

Electronic searches were carried out in PubMed (MEDLINE), Scopus, Web of Science, and Embase databases using the following keywords: (“salivary gland” OR “salivary glands”) AND (tumor OR tumors OR tumour OR tumours OR neoplasm OR neoplasms OR cancer OR cancers OR carcinoma OR carcinomas) AND (infant OR infants OR newborn OR newborns OR child OR child OR pediatric OR pediatrics OR paediatric OR paediatrics OR adolescent OR adolescents OR adolescence OR youth OR youths OR teen OR teens OR teenager OR teenagers). Articles that evaluated clinicopathological features of primary epithelial salivary gland tumors in pediatric patients (0 to 19 years) published in the English-language literature were assessed.

## Results

**Figure 1:**
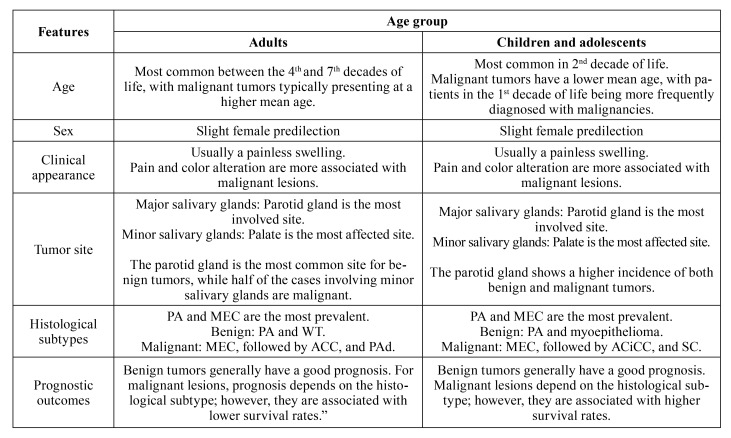
Histopathological features of most common salivary gland tumors in pediatric patients. Pleomorphic adenoma showing myoepithelial and epithelial cells immersed in a chondromyxoid matrix (A, hematoxylin-eosin [HE] 100x) and fibrous stroma (B, HE 100x). Some duct structures filled with eosinophilic secretion are also observed. Low-grade mucoepidermoid carcinoma exhibiting mucous, epidermoid and intermediate cells lining cystic like spaces. Some clear cells are also noted (C, HE 100x). High grade mucoepidermoid carcinoma showing epidermoid cells with significant nuclear pleomorphism. Some mucous cells are observed in the lower right (D, HE 200x).

SGTs in pediatric patients are slightly more prevalent in females and typically arise during the second decade of life. The parotid gland is the most commonly affected site for both benign and malignant tumors, which usually present as an asymptomatic mass. Pleomorphic adenoma and mucoepidermoid carcinoma are the most frequently observed entities. Surgical intervention remains the primary treatment modality, and the overall prognosis is generally favorable. Table 1 summarizes the main characteristics of SGTs in adult and pediatric patients, and Fig. 1 illustrates the main histopathological diagnoses of SGTs in children and adolescents.

## Discussion

SGTs are also uncommon in children and adolescents, representing less than 5% of all head and neck tumors in this age group (1,9). In the Chinese population, pediatric SGTs constitute 7.5% of all cases (10). In Brazil, the occurrence ranges from 4.1% to 10.1% (11,12). In the United States, this prevalence is even lower, varying from 1.6% to 3.6% (13,14). A study conducted in Africa reported a prevalence of 7.8% (8). Conversely, European countries tend to report lower frequencies, with approximately 2.7% observed in pediatric patients (15).

A previous systematic review conducted by our group (9) identified a total of 2,937 cases from 54 studies of pediatric SGTs. Out of these 75.4% were malignant tumors and 24.6% cases were benign tumors; however, most of the included studies were conducted in tertiary health centers (77.8%) leading a publication bias (9). Overall, SGTs are more slightly prevalent in females (57.1%), with a mean age of 13.3 years. The parotid gland is the most affected location (81.9%), followed by the submandibular gland (11.5%) and palate (4.5%). Similar to adults, sublingual gland is also an extremely uncommon location for SGTs. Among the cases with the description of the clinical presentation, swelling in the affected gland is the most reported finding (99.2%) (9).

Another study conducted by our group (16), which examined the clinicopathological profile of 203 pediatric patients with epithelial SGTs from Brazil, South Africa, and the United Kingdom, revealed a comparable distribution of benign (61.6%) and malignant tumors (38.4%) when contrasted with adult cases. In the following sections, we will describe the main characteristics of benign and malignant SGTs in children and adolescents separately.

- Benign SGTs

A female predominance in the incidence of benign SGTs has been reported, with approximately 90% of cases occurring in the second decade of life. Generally, benign tumors affect patients with a higher average age. It is important to mention the significant heterogeneity in age limits described across different studies. While most set the upper limit at 18 or 19 years, some define it as 14 years. These discrepancies arise from confusion regarding the boundaries of the pediatric age group, which can significantly impact epidemiological analyses (9,11,16,17).

The proportion of major and minor SGTs varies across studies, depending on the types of services analyzed. Oral pathology laboratories tend to report a higher frequency of minor SGTs, while hospitals generally have more cases involving major salivary glands. Although most published cases originate from tertiary health services, the parotid gland is identified as the most affected site, followed by submandibular and palate. The buccal mucosa and lips are other common sites for benign minor SGTs. Clinically, benign tumors typically present as slow-growing, painless nodule with a smooth surface and normal color. Thus, the clinical features of benign SGTs in the pediatric population are generally similar to those observed in adults (9,16,18-20).

Regarding histopathological diagnoses, PA is the most prevalent neoplasm. Notably, Warthin tumor, often reported as the second most common benign SGT in adults is not usually diagnosed in children and adolescents (9,16,20-22). This discrepancy may be attributed to the fact that nearly all adult patients diagnosed with Warthin tumor have a history of cigarette smoking (23).

Similar to adults, the primary treatment option for benign SGTs is surgical excision. The recurrence rate is approximately 10% and may occur following incomplete excision. In contrast, malignant transformation of pleomorphic adenoma (PA) has not been reported in pediatric patients (9,16,20).

- Malignant SGTs

A retrospective study conducted in a Brazilian cancer center by Arboleda and colleagues (24), identified 11 (3.0%) cases of salivary gland carcinomas out of 367 pediatric head and neck malignancies, with MEC the most frequent followed by acinic cell carcinoma (AciCC). Although a systematic review (9) found a higher prevalence of malignant SGTs, it is important to highlight that the majority of published cases is from tertiary health center, of which could lead a publication bias and not represent a real epidemiology. In this scenario, Quixabeira *et al* (16) analysed 203 cases of SGTs in pediatric patients and included 8 oral pathology laboratories (141 cases) and 3 oncology hospital (62 cases). Out of these, 78 were malignant, with MEC (54 cases; 69.2%) the most common subtype followed by AciCC (9 cases; 11.5%), and secretory carcinoma (6 cases; 7.7%).

When analyzing children and adolescents diagnosed with malignant SGTs, about 80% of cases occur in the second decade of life (9,11,16,20). However, these malignant tumors are diagnosed at a younger average age of 12.8 years, compared to 14.2 years for benign tumors, with a noTable number of cases emerging in patients under 10 years old. Still, a recent study conducted by Jesberg *et al* (20) in the United States found no significant age difference between benign and malignant tumors (13.6 years versus 13.0 years) but tumors in females were more frequently malignant (M:F 1:1.3 vs. 1:2.7 for benign and malignant tumors, respectively). In adult population, the parotid gland accounts for more than half of SGT cases, and minor salivary glands are a common site for salivary carcinomas (1,4,5). In contrast, among pediatric patients, the parotid gland is the predominant site for both benign and malignant tumors (9). Even though, most published cases are originated from oncology hospitals, which tend to receive a higher number of cases affecting the major salivary glands, Quixabeira *et al* (16) analyzed both oral pathology laboratories and oncology hospitals, finding that the parotid gland still accounted for a significant number of malignant cases. This emphasizes a significant role of the parotid gland in SGTs in this age group.

Clinically, benign and malignant SGTs often exhibit overlapping features, making differentiation challenging. However, the presence of pain and color alterations can serve as indicators of malignancy, as observed in adults (6,9). Recognizing these symptoms is crucial for determining whether to proceed with an incisional or excisional biopsy, as timely and appropriate intervention can significantly impact patient outcomes.

Regarding histological subtypes, MEC is the most common malignant SGT. Although MEC is the predominant salivary gland carcinoma in adults, other common entities such as polymorphous adenocarcinoma and ACC are less frequently observed in pediatric patients (1,4,5,9). Notably, AciCC and secretory carcinoma is the second and third most frequent malignant SGT in this age group, respectively (9,16,20,25). This highlights the differences in tumor prevalence and behavior between adult and pediatric populations.

Secretory carcinoma was first described in 2010, mimicking AciCC. Then, studies with AciCC cases published prior to 2010 should be interpreted with caution, as some of these cases may need re-evaluation for the presence of ETV6 translocation and if positive, reclassified as secretory carcinoma (25,26).

Additionally, it is important to recognize a rare malignant neoplasm primarily diagnosed in the neonatal period or early childhood known as sialoblastoma. The parotid gland is the most common site for this tumor. Although sialoblastomas generally have a good prognosis, they are associated with a significant recurrence rate of 35.7% (27).

SGTs in children and adolescents often exhibit more favorable characteristics compared to those in adults. The overall survival rate for this age group is approximately 85%, with most cases diagnosed as low-grade MECs. It is well known that the behavior of malignant SGTs varies based on histological (9,16,28,29). Low-grade MEC and polymorphous adenocarcinoma typically present a good prognosis, while high-grade MEC, ACC and AciCC are associated with lower survival rates and a higher potential for metastasis and recurrence (30,31). This underscores the importance of accurate histological classification in guiding treatment and management strategies.

Molecular alterations in salivary gland carcinomas have been studied, including the CRTC1-MAML2 and MYB-NFIB fusion genes found in MEC and ACC, respectively (32). The frequency of CRTC1-MAML2 translocation ranges from 33.7% to 69.7% and is associated with low-grade tumors and younger patients (33). Notably, all 12 pediatric MEC cases analyzed by Techavichit *et al* (34) and 15 out of 17 pediatric MEC cases examined by Locati *et al* (35) tested positive for the MAML2 fusion. Therefore, further research is necessary to evaluate molecular findings and their prognostic implications for malignant salivary gland tumors in this age group compared to adults.

As in adults, SGTs there are currently no effective systemic therapies (36), making surgery the primary treatment option for pediatric malignant cases. Some authors advocate for postoperative radiotherapy for cases exhibiting some aggressive features, including lymph node involvement, perineural and vascular invasion, as well as soft tissue extension (17,37).

Additionally, fine-needle aspiration cytology (FNAC) is the standard diagnostic approach for preoperative assessment of salivary gland tumors in adults, as endorsed by consensus guidelines. However, its effectiveness in pediatric patients remains debated due to a lack of data and challenges such as patient cooperation, the need for sedation, and potential procedural risks. Recent studies, however, have highlighted FNAC's excellent diagnostic performance in distinguishing malignant from benign pediatric salivary gland lesions. FNAC is generally safe, well-accepted by pediatric patients, and can often be performed in an outpatient setting without sedation (38-40).

In summary, SGTs in pediatric patients are rare and show a slight predilection for females, typically arising during the second decade of life. These tumors most commonly affect the parotid gland and palate, usually presenting as asymptomatic, slow-growing masses. Although publication bias makes it difficult to determine whether benign SGTs are more prevalent than malignant tumors, PA and MEC are recognized as the most common entities. The parotid gland remains the primary site involved for both benign and malignant SGTs. Surgical intervention is the main treatment approach, and the overall prognosis is generally favorable.

Considering the limitations identified in the published studies, we propose the following recommendations for future reports on SGTs in pediatric patients:

1. We suggest following the WHO age definition for pediatric patients (children 0-9 years, and adolescents 10-19 years) when reporting or assessing SGTs in research papers to facilitate a better analysis of epidemiological data;

2. Clarify whether the pediatric population has a higher frequency of salivary gland carcinomas by including data from various types of services, such as oral pathology laboratories and oncological hospitals;

3. Evaluate prognostic outcomes of SGTs in children and adolescents by providing clear information on epidemiological, clinical, and follow-up data;

4. Investigate molecular findings in pediatric SGTs to address the significance of molecular changes in this age group compared to adults.

## Figures and Tables

**Table 1:** Comparative overview of mean characteristics of epithelial salivary gland neoplasms in adult and pediatric patients.

Features	Age group	
	Adults	Children and adolescents
Age	Most common between the 4^th ^and 7^th^ decades of life, with malignant tumors typically presenting at a higher mean age.	Most common in 2^nd^ decade of life. Malignant tumors have a lower mean age, with patients in the 1^st^ decade of life being more frequently diagnosed with malignancies.
Sex	Slight female predilection	Slight female predilection
Clinical appearance	Usually a painless swelling. Pain and color alteration are more associated with malignant lesions.	Usually a painless swelling. Pain and color alteration are more associated with malignant lesions.
Tumor site	Major salivary glands: Parotid gland is the most involved site. Minor salivary glands: Palate is the most affected site. The parotid gland is the most common site for benign tumors, while half of the cases involving minor salivary glands are malignant.	Major salivary glands: Parotid gland is the most involved site. Minor salivary glands: Palate is the most affected site. The parotid gland shows a higher incidence of both benign and malignant tumors.
Histological subtypes	PA and MEC are the most prevalent. Benign: PA and WT. Malignant: MEC, followed by ACC, and PAd.	PA and MEC are the most prevalent. Benign: PA and myoepithelioma. Malignant: MEC, followed by ACiCC, and SC.
Prognostic outcomes	Benign tumors generally have a good prognosis. For malignant lesions, prognosis depends on the histological subtype; however, they are associated with lower survival rates.	Benign tumors generally have a good prognosis. Malignant lesions depend on the histological subtype; however, they are associated with higher survival rates.

PA - pleomorphic adenoma; WT - Warthin Tumor; MEC - mucoepidermoid carcinoma; ACC - adenoid cystic carcinoma; PAd - polymorphous adenocarcinoma; ACiCC - acinic cell carcinoma; SC - secretory carcinoma.
